# Use pattern of maternal health services and determinants of skilled care during delivery in Southern Tanzania: implications for achievement of MDG-5 targets

**DOI:** 10.1186/1471-2393-7-29

**Published:** 2007-12-06

**Authors:** Rose NM Mpembeni, Japhet Z Killewo, Melkzedeck T Leshabari, Siriel N Massawe, Albrecht Jahn, Declare Mushi, Hassan Mwakipa

**Affiliations:** 1 School of Public Health and Social Sciences, Department of Epidemiology and Biostatistics, Muhimbili University of Health and Allied Sciences, P.O. Box 65015, Dar-Es-Salaam, Tanzania; 2 Department of Behavioural Sciences, School of Public Health and Social Sciences, Muhimbili University of Health and Allied Sciences, P.O.Box 65015, Dar-Es-Salaam, Tanzania; 3 Department of Obstetrics and Gynaecology, School Of Medicine, Muhimbili University of Health and Allied Sciences, P.O. Box 65001, Dar-Es-Salaam, Tanzania; 4 Department of Tropical Hygiene and Public Health, Heidelberg University, Im Neuenheimer Feld 324, 69120, Heidelberg, Germany; 5 Mtwara District Council, P.O. Box 524, Mtwara, Tanzania

## Abstract

**Background:**

Almost two decades since the initiation of the Safe motherhood Initiative, Maternal Mortality is still soaring high in most developing countries. In 2000 WHO estimated a life time risk of a maternal death of 1 in 16 in Sub- Saharan Africa while it was only 1 in 2800 in developed countries. This huge discrepancy in the rate of maternal deaths is due to differences in access and use of maternal health care services. It is known that having a skilled attendant at every delivery can lead to marked reductions in maternal mortality. For this reason, the proportion of births attended by skilled health personnel is one of the indicators used to monitor progress towards the achievement of the MDG-5 of improving maternal health.

**Methods:**

Cross sectional study which employed quantitative research methods.

**Results:**

We interviewed 974 women who gave birth within one year prior to the survey. Although almost all (99.8%) attended ANC at least once during their last pregnancy, only 46.7% reported to deliver in a health facility and only 44.5% were assisted during delivery by a skilled attendant. Distance to the health facility (OR = 4.09 (2.72–6.16)), discussion with the male partner on place of delivery (OR = 2.37(1.75–3.22)), advise to deliver in a health facility during ANC (OR = 1.43 (1.25–2.63)) and knowledge of pregnancy risk factors (OR 2.95 (1.65–5.25)) showed significant association with use of skilled care at delivery even after controlling for confounding factors.

**Conclusion:**

Use of skilled care during delivery in this district is below the target set by ICPD + of attaining 80% of deliveries attended by skilled personnel by 2005. We recommend the following in order to increase the pace towards achieving the MDG targets: to improve coverage of health facilities, raising awareness for both men and women on danger signs during pregnancy/delivery and strengthening counseling on facility delivery and individual birth preparedness.

## Background

It is almost two decades now since the initiation of the Safe Motherhood Initiative, but maternal mortality is still soaring high in most of the developing countries. In the year 2000, WHO estimated maternal mortality ratio of 920/100000 live births for Sub-Saharan Africa with a lifetime risk of a maternal death of 1 in 16. These rates are very high when compared to the developed countries lifetime risk of 1 in 2800 estimated during the same time period [1].

Reports show that more than three quarters of maternal deaths are due to direct obstetric causes such as haemorrhage, abortion, sepsis, ruptured uterus and hypertensive diseases of pregnancy [2]. Most of these maternal deaths can be averted because the technical and political means to prevent them have been available for many decades. For example, it is known that having a skilled attendant at every delivery can lead to marked reductions in maternal mortality and morbidity [3-6]. In the technical consultation held ten years of Safe motherhood initiative it was clearly stated that: "Having a health worker with midwifery skills present at child birth, backed up with transport in case of emergency referral is perhaps the most critical intervention for making motherhood safe" [2]. Due to this fact, the proportion of births attended by skilled health personnel is used as one of the important indicators to monitor progress towards the achievement of the millennium development goal of reducing maternal mortality ratios. The targets set at the International Conference on Population and Development+5 (ICPD+) is to have more than 80% of deliveries assisted by skilled attendants globally by 2005, 85% by 2010 and 90% by 2015 [7].

In Tanzania, like other Sub Saharan Africa countries, maternal mortality remains to be a problem of public health importance. The 2004 Tanzania Demographic and Health Survey (TDHS) published a maternal mortality ratio (MMR) of 578/100000 live births [8] but other community based studies found MMR as high as 990/100000 live births in some districts [9]. The life time risk of a maternal death in Tanzania has been estimated at 1 in 38 [10]. Tanzania was ranked 6^th ^among the 13 countries with highest levels of maternal mortality which account for 67% of all world maternal deaths [1].

Use of health facilities for delivery is still very low in Tanzania. It is reported that only 47% of deliveries occur in the health facilities and the remaining more than half deliver at home assisted by unskilled attendants [8]. This is happening amidst the fact that Tanzania has a good network of health facilities with about 72% of the population residing within 5 km and 90% reside within 10 km of a health facility. Also maternal health care services are provided free of charge in almost all public facilities.

The objective of this study was to assess the use determinants of skilled attendants at delivery in Mtwara rural district. The information obtained will help the district health management team to develop interventions to improve use of delivery care services and ultimately achieve the millennium goal to reduce the high rates of maternal mortality.

## Methods

### Study Design

The study was a cross sectional study. Quantitative research methods were employed in the study which involved interviews to a random sample of women (age 14–50) who gave birth within one year prior to the survey using a structured questionnaire. The questionnaire was pre-tested in a similar population in a neighbouring district to test for clarity, validity and reliability of the questions after which the tool was revised accordingly and finalised for use.

### Study Site

The study was conducted in the Mtwara rural district. Mtwara rural, one of the five districts that make up Mtwara region is located in the South Eastern corner of Tanzania. The district has a total population of 204,770 [11] and has a total of 34 health facilities, 4 being health centres and 30 being dispensaries. Normally at the dispensary the staff should include a clinical officer (Certificate holder in clinical medicine) and a Maternal and Child Health Aid (MCHA) while in the health centre there should be a Clinical Officer and 2 nurse midwives. Both health centres and dispensaries are supposed to provide basic emergency obstetric care services but sometimes not all the six core functions are available. The district has no hospital but the regional hospital (Ligula) serves as the first referral level for emergency obstetric care for this district where emergency obstetric care services are provided for 24 hrs. Few villages are located more than 80 km from the regional hospital but the majority of the populations is within 60 kilometres. The district has one ambulance stationed at the district headquarters and all health centres and 5 distant dispensaries were fitted with radio calls for communication in case of an emergency. The dispensaries in the district were recently provided with what are locally called cycle ambulances (bicycles fitted with locally made stretcher). During an emergency, relatives pick the cycle from the dispensary and use it to transport the patient to the dispensary or even to the hospital. Both the ambulance and the cycle ambulances are used free of charge. The district has a high maternal mortality ratio estimated at 600 per 100,000 live births and only a small proportion of women use of modern contraceptives (25%).

### Sampling and Sample size

This study was conducted as a baseline survey of an intervention study aimed at increasing skilled attendance during delivery and increasing referral compliance. A multistage cluster random sampling was employed to select the study sample. We first selected a random sample of 24 health facilities using simple random sampling technique. For each of the selected health facility, one village in its catchment area was selected randomly. In the selected village, a house to house survey was conducted and all women who had given birth within the previous one year were interviewed.

### Data Analysis

Data entry and cleaning was done using EPI Info 6.04d program while data analysis was done using SPSS for Windows Version 11. A composite socio-economic status indicator (wealth index) was created using information on source of drinking water, type of toilet facilities, housing construction material, household assets, ownership of any form of transportation, ownership of animals, land ownership and source of family income. Data Reduction using the principle components and factor analysis was used to generate weighted scores from the above variables and normalized with a mean of zero and standard deviation of one. The resulting scores were then summed up within households, ranked and used to stratify the households into 5 levels of socio-economic status.

A variable, knowledge of pregnancy danger signs, was arrived at but analyzing the number of danger signs the respondent mentioned spontaneously. Those who mentioned none were considered to have no knowledge, those respondents who mentioned up to three danger signs were considered to have low knowledge and those who mentioned 4 or more danger signs were considered to have moderate knowledge of pregnancy danger signs. None of the respondents was considered to have a high knowledge as none mentioned more than 8 out of a total of 17 risk factors printed on the antenatal card.

The χ^2 ^test was used to assess association between use of maternal health care services and socio-demographic variables, and other service characteristics. P-values of less than 0.05 were considered significant.

Multiple logistic regression was used to assess individual effect of variables on use of skilled care attendance while adjusting for potential confounding variables.

## Results

A total of 974 women who gave birth within the 1 year prior to the survey were interviewed. Their ages ranged from 14 to 50 years with mean age of 26.86 ± 6.76. One hundred and four women (10.7%) did not know their ages (Table 1).

**Table 1:** Socio-demographic characteristics of the study Sample

Variable	N (%)
**Age**	
14–19	120 (12.3)
20–34	624 (64.0)
35+	127 (13.0)
Age not reported	103 (10.7)
**Years spent in school**	
0	482 (49.4)
1–6	125 (12.8)
7+	367 (37.8)
**Marital status**	
Single	98 (10.1)
Married	752 (77.2)
Divorced/Widowed/separated	124 (12.7)
**Household size**	
0–5	632 (64.9)
6–11	334 (34.3)
Missing	8 (0.8)
**Parity**	
1–4	793 (81.4)
5+	181 (18.6)
**Distance to facility (km)**	
0–5	791 (81.2)
>5	183 (18.8)
**Socio-Economic Status**	
Very Low	190 (19.5)
Low	191 (19.6)
Medium	187 (19.2)
High	189 (19.4)
Very high	189 (19.4)
Missing	28 (2.9)
**Total**	974 (100)

Almost half (49.4%) of the respondents had never been to school and of those who reported to have attended formal schools, almost three quarters (72.2%) reported only seven years of schooling. An overwhelming majority of respondents (94%) were Muslims and of Makonde tribe. Major occupation of respondents was subsistence farming, accounting for 95.7% of the respondents. Over three quarters (77.2%) were married or cohabiting but also a significant proportion (12.2%) were divorced (Table 1). Number of children ever born ranged from 1 to 11 with a median of 3.

### Use of antenatal care (ANC) services

Respondents were asked whether they attended antenatal clinics during their most recent pregnancy and for those who attended, information on age at start of ANC and the number of ANC visits was obtained. Findings showed that utilization of antenatal care services was universal with 972 (99.8%) of respondents reporting to have attended antenatal clinic at least once during their last pregnancy. At the ANC clinic women get screened for risk factors and receive appropriate advices, TT vaccinations, health education and counseling on Individual birth planning, Intermittent presumptive treatment of malaria and iron supplementation.

### Use of skilled care at delivery

**Figure 1:**
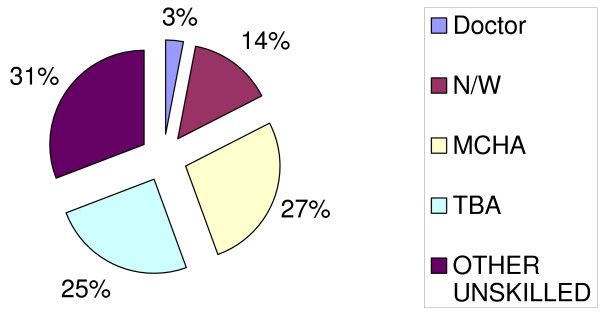
Qualifications of delivery attendants.

Less than half, (46.7%) of respondents reported to have delivered in a health facility in their most recent delivery. Of these, 35% delivered in a hospital level and 65% delivered in dispensaries or in health centers. Skilled attendance at delivery was reported by only 433 (44.5%) of the respondents and of these 16 (3.6%) were home deliveries. Maternal and Child Health Aids (MCHA) assisted over a quarter (27.1%) of all deliveries while nurse midwifes attended only 14.4% of the deliveries. Among women who delivered at home, 50.1% were assisted by untrained relatives or friends while 46.3% were assisted by Traditional Birth Attendants (TBA's) and 3.6% were assisted by skilled midwives (Fig 1).

### Determinants of skilled care at delivery

Table 2 shows that proportion of women who were attended during delivery by a skilled attendant was seen to decrease significantly with increasing age of women from 57.5% among women below 20 years of age to only 48.8% among women aged 35 or more years (p < 0.01). Years spent in school also showed a significant association with seeking of skilled care during delivery with women who have more schooling years having a higher proportion of deliveries (50.4%) attended by skilled personnel compared to those with fewer schooling years or those who did not go to formal schooling (p < 0.01). A significantly higher proportion (57.1%) of women who are single delivered with a skilled attendant compared to their married counterparts (41.8%) (p < 0.01). The proportion of women with skilled attendants at delivery was also seen to decrease with increasing distance to the health facility which provide delivery care from 50.1% among women residing within 5 km of a health facility to only 20.2% among those residing more than 5 km from a health facility (OR 3.84 (95%CI 2.61–5.65) (Table 2).

**Table 2:** Determinants of skilled attendance at delivery.

**Variable**	**N (%)**	**% with skilled care**	**Unadjusted OR (95%CI)**	**Adjusted OR (95% CI)**
**Distance to the facility(km)**				
0–5	791 (81.2)	50.1	3.84(2.61–5.65)	4.09 (2.72–6.16)
6+	183 (18.8)	20.2***	1	
**Age (yrs)**				
<20	120 (12.3)	57.5	1.86 (1.25–2.76)	
20–34	624 (64.1)	42.6	1	
35 +	127 (13.0)	48.8	1.27 (0.87–1.86)	
Unknown Age	103 (10.7)	35.0		
**Years spent in school**				
0	482 (49.5)	39.4	1	1
1–6	125 (12.8)	46.4	1.27 (0.85–1.89)	1.04 (0.66–1.64)
7+	367 (37.7)	50.4**	1.55 (1.18–2.05)	1.44 (1.05–1.96)
**Marital status**				
Single	98 (10.1)	57.1	1	
Married	752 (77.2)	41.8	0.52 (0.34 – 0.8)	0.41 (0.25–0.66)
Divorced/widowed/separated	124 (12.7)	50.8**	0.75 (0.44 – 1.28)	0.76 (0.42–1.37)
**Household size**				
0–5	632 (64.9)	44.9	1	
6–11	334 (34.3)	43.7	0.94 (0.72–1.22)	
Missing	8 (0.8)			
**Parity**				
1–4	793 (81.4)	45.5	1	
5+	181 (18.6)	39.8	0.77 (0.55–1.07)	
**Timing of ANC first visit**				
1–5 months	410	49.3	1	
6+ months	551	40.8	1.001 (0.99–1.01)	
Missing	13			
**Number of ANC visits**				
1.3	331	37.8	1	1
4+	623	48.8**	1.54 (1.17–2.02)	1.43 (1.07–1.93)
Missing	20			
**Discussed with partner on where to deliver**				
No	582 (59.7)	36.3	1	
Yes	385 (39.5)	56.1***	2.27 (1.74–2.95)	2.37 (1.75–3.22)
Missing	7 (0.7)			
**Advised where to go for delivery during ANC**				
No	198 (20.3)	28.3	1	
Yes	772 (79.2)	48.4***	2.31 (1.64–3.25)	1.82 (1.25–2.63)
Missing	4 (0.4)			
**Socio Economic status**				
Very low	190 (19.5)	41.1	1	
Low	191 (19.6)	37.2	0.83 (0.55–1.26)	
Moderate	187 (19.2)	42.2	1.01 (0.67–1.52)	
High	189 (19.4)	47.6	1.28 (0.85–1.92)	
Very high	189 (19.4)	52.9	1.65 (0.75–3.67)	
Missing	28 (2.9)	53.6		
**Knowledge of risk factors**				
No knowledge	621 (63.8)	39.0	1	1
Low knowledge	281 (28.9)	50.5	1.65 (1.25–2.20)	1.29 (0.93–1.79)
Moderate knowledge	72 (7.4)	68.1***	3.24 (1.93–5.43)	2.95 (1.65–5.25)

*p < 0.05, **p < 0.01 ***p < 0.001

Women who reported that they ever discussed with their husbands or partners on where to go for delivery while pregnant and those who were advised during ANC by health workers to deliver in a health facility had a higher proportion delivering with a skilled attendant compared to those who were not (p < 0.05) (Table 2). Proportion of women with skilled care at delivery increased with knowledge of danger signs from 39% among women who did not mention any to 68% among those who mentioned 4 or more danger signs (p < 0.05). Women who reported that they had health problems during pregnancy did not have an increased chance to use skilled attendants for delivery compared to those who had no health problems during pregnancy (p > 0.05). Women who started ANC clinic early and those who had 4 or more ANC visits were more likely to be assisted during delivery by a skilled attendant compared to those who booked late for ANC and those with fewer than recommended number of ANC visits.(Table 2).

All variables which showed a significant association with type of delivery care in the bivariate analysis were put in a multiple logistic regression model to assess individual variable effects on skilled care during delivery (Table 2).

Even after controlling for other variables, distance to the health facility providing delivery care OR = 4.09 (95% CI 2.72–6.16), discussion with male partners on place of delivery OR = 2.37 (95% CI 1.75–3.22), advice on place of delivery during ANC OR = 1.82 (95% CI 1.25–2.63), number of ANC visits OR 1.43 (95% CI 1.07–1.93) and knowledge of risk factors OR = 2.95 (95% CI 1.65–5.25) were seen to be significantly associated with use of skilled care at delivery (Table 2).

## Discussion

Antenatal care especially when thought early allows regular checkups for the health of the pregnant woman and early interventions incase of any complications. Findings of this study show that almost all women (99%) sought antenatal care at least once during their last pregnancy. These findings compare well with those of the Tanzania Demographic and Health Surveys [8]. Main reasons reported earlier for this universal ANC attendance included seeking for immunizations for tetanus, prophylaxis for anaemia and malaria and the fact that women want to be assured that their unborn babies are well [12,13]. Majority of women delayed their first ANC visit and about a third went for fewer than recommended number of visits. Late start of ANC and too few visits reduces the effectiveness of the ANC as some necessary interventions are started either very late in pregnancy or they are not completed before the time of delivery. These results also agree with a study conducted in Nigeria, where the median time for first ANC visit was found to be 23.7 weeks [14]. Women book late to avoid going for many ANC visits particularly in circumstances where the facility is quite far and there are no reliable means of transport.

Proportion of women who delivered in the health facility was only 46.7% where as the proportion who had skilled assistance was 44.5%. These estimate compares well with the national level rates (47% Vs 46%) observed in the most recent demographic and Health survey but higher compared to the rates obtained from the study region (37%) in the same survey [8]. The low coverage rate in the TDHS sample in this region may be the cause of these discrepancies. Low rates of health facility deliveries in developing countries have been reported by several other researchers [15-20]. Fear of being referred to hospital, availability of TBA's, emergency nature of labour were mentioned to be the major causes of home deliveries. This low utilization of skilled attendants at delivery represents the great challenge to achieve the MDG's in the next nine years.

A number of socio demographic and economic factors were found to have a significant influence on use of skilled care at delivery. They include women's age, education level, and marital status. Younger women are just starting child bearing and are told to be in a high risk group and so they tend to fear home deliveries. It is also possible that the new generation with a higher proportion of women who have formal education have different perspectives on delivery care when compared to the older generations. These variables also influence the status of the woman in the society which has been found to influence decision making. A woman who is educated, single and of higher socio economic status is able to make wise decisions about her own health than their counterparts. Similar findings were reported by previous researchers [16,18,19,21,22]. It has been reported that both economic and social dimensions of the distribution of power between spouses influence the use of services [23].

Women who were knowledgeable of risk factors were more likely to utilize health facilities for delivery compared to those with no knowledge. Similar findings were reported in Malawi and in Zambia [18,24]. It is expected that a better informed individual is better placed to make reasonable decisions.

Distance to the health facility was a significant determinant of type of delivery care. This was said to be made worse by the fact that there are no means of transport to the facility. Similar findings were reported by a number of researchers previously [16,18,25].

Women who had more than 4 ANC visits were more likely to deliver with a skilled attendant than those with fewer visits. Similar findings were reported in Cambodia [16,26]. This may be due to the fact that women with more ANC visits also showed a higher satisfaction with the care quality and hence more likely to use health services for delivery. It is also a fact that many ANC visits expose the women to more health education and counseling which are both likely to increase service utilization. This finding lead previously to a recommendation that although antenatal care may not be efficient in identifying women who are most in need of obstetric care, if promoted it may become an effective instrument to facilitate better use of emergency obstetric care services [26].

Surprisingly women who reported illnesses/pregnancy complications during pregnancy did not have an increased chance to use health facilities. This may be due to wrong perceptions of causation which lead them to seek care from traditional healers instead of modern health facilities.

In contrast to findings of several other studies, perceived quality of ANC care was not seen to influence the choice of a delivery attendant. This may be due to the fact that majority (85%) of women in this study perceived the ANC care quality as good.

## Conclusion

In the study area, although almost all pregnant women seek antenatal care, less than half of deliveries are attended by skilled personnel. The proportion of births attended by skilled personnel is far below the ICPD+ target of having 80% births attended by skilled attendants by the year 2005. Distance to the facility, advice by a health worker on place of delivery, knowledge of danger signs and a higher number of ANC visits were found to be significantly associated with the type of delivery attendant.

We recommend the following in order to increase our pace towards the millennium development goal targets: To improving coverage of health facilities which provide skilled delivery care, To raise the status of women in terms of education and socio-economic status, and to improve provision of health education to women especially on danger signs during pregnancy and delivery and also intensify individual counselling of women on hospital delivery and on individual birth preparedness.

## Competing interests

The author(s) declare that they have no competing interests.

## Authors' contributions

RNMM designed the study, participated in data collection, analysis and drafted the manuscript for publication. JZK, MTL, SNM and JA provided scientific advices on the design of the study, data analysis and through out the preparation of the manuscript, DM and HM were involved in the design, data collection and preparation of manuscript. All authors read and approved the manuscript.

## Pre-publication history

The pre-publication history for this paper can be accessed here:


